# Supplementary material for: Splice-Junction-Based Mapping of Alternative Isoforms in the Human Proteome
Source: Cell Rep. Author manuscript; Available in PMC 2020 Jan 15. (PMC6961840; doi:10.1016/j.celrep.2019.11.026)
Supplement: 3 [file NIHMS1546469-supplement-3.zip › DF2/PXD009737/TestisGluC-18-Q9HD26-LHAKTGQSADSGTIKAKLE.pdf]

A

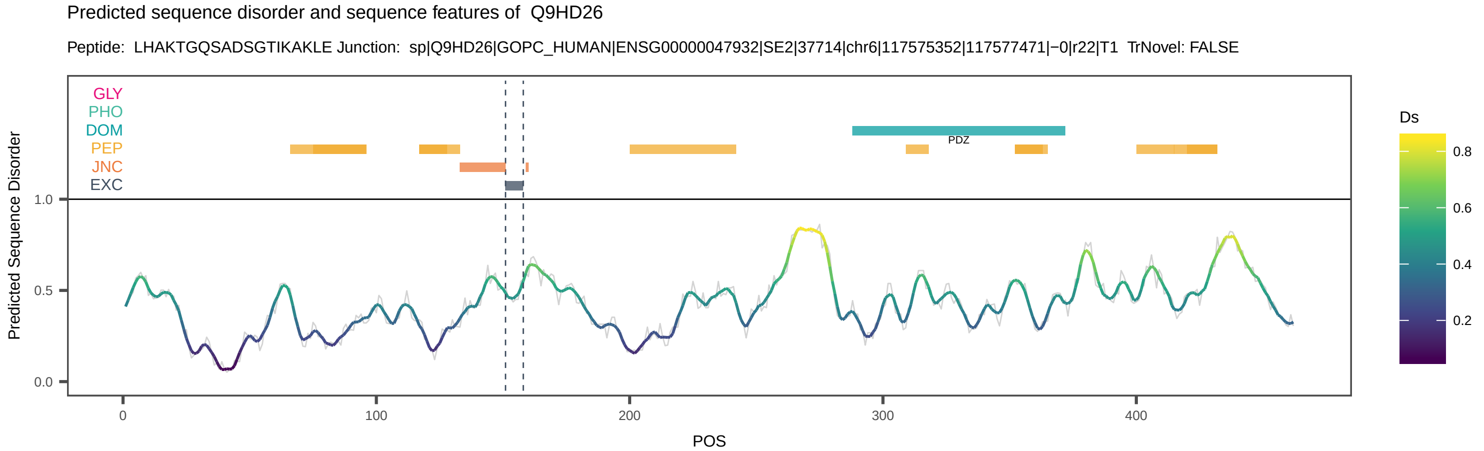
Predicted sequence disorder and sequence features of Q9HD26
Peptide: LHAKTGQSADSGTIKAKLE Junction: sp|Q9HD26|GOPC_HUMAN|ENSG00000047932|SE2|37714|chr6|117575352|117577471|−0|r22|T1 TrNovel: FALSE
GLY
PHO
DOM
PEP
JNC
EXC
PDZ
Predicted Sequence Disorder
1.0
0.5
0.0
0
100
200
300
400
POS
Ds
0.8
0.6
0.4
0.2

B

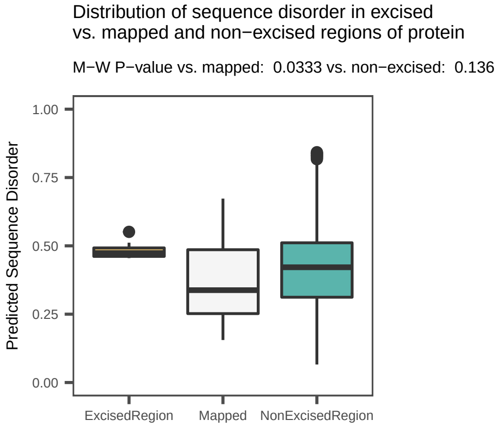
Distribution of sequence disorder in excised
vs. mapped and non−excised regions of protein
M−W P−value vs. mapped: 0.0333 vs. non−excised: 0.136
Predicted Sequence Disorder
1.00
0.75
0.50
0.25
0.00
ExcisedRegion
Mapped
NonExcisedRegion

C
